# Preparation and Solid-State Characterization of Eltrombopag Crystal Phases

**DOI:** 10.3390/molecules26010065

**Published:** 2020-12-25

**Authors:** Vincenzo Mirco Abbinante, Massimo Zampieri, Giuseppe Barreca, Norberto Masciocchi

**Affiliations:** 1 Dipartimento di Scienza e Alta Tecnologia & To.Sca.Lab., Università dell’Insubria, via Valleggio 11, 22100 Como, Italy; vmabbinante@uninsubria.it; 2 Chemessentia srl, via Bovio 6, 28100 Novara, Italy; giuseppe.barreca@chemessentia.it

**Keywords:** Eltrombopag, polymorphism, X-ray diffraction, thermal analysis, pattern indexing

## Abstract

Eltrombopag, of C_25_H_22_N_4_O_4_ chemical formula, is a drug used against thrombocytopenia, marketed worldwide under different tradenames in the form of its bis-olamine salt. The free acid (CAS no. 496775-61-2) is an intermediate species used for the final drug isolation and is reported to crystallize in more than 20 distinct crystal forms, including a large number of hydrates and solvates. Their identification, and, ultimately, their quantification in industrial lots require the usage of accurately measured X-ray powder diffraction pattern, as well as the assessment of the metrical features (crystal symmetry and lattice parameters), nowadays accessible by powerful crystallographic software. Here, the complete indexing of 13 monophasic samples, prepared using literature or newly tailored crystallization methods, jointly to simultaneous thermogravimetric and calorimetric analyses and to variable temperature X-ray diffraction studies, provide a clear picture of the stability fields of the different crystal phases and their mutual interconversion processes, leading, in a few cases, to new and unexpected crystalline polymorphs or solvates of the pristine unsolvated Form I.

## 1. Introduction

**Scheme 1:**
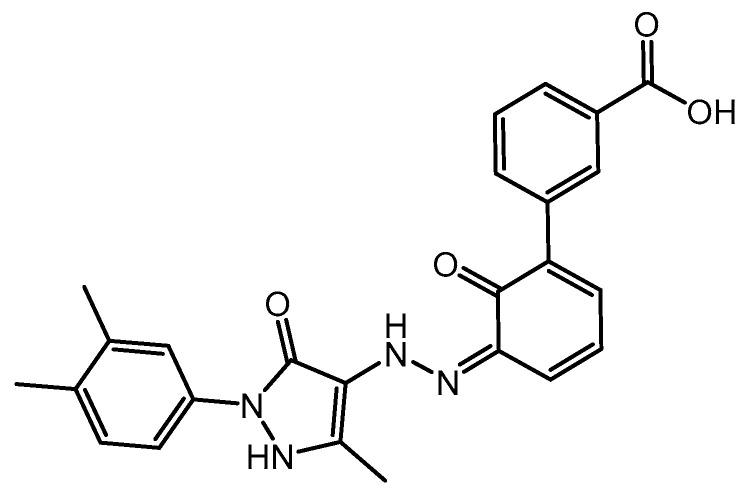
Sketch of the Eltrombopag molecular connectivity.

Eltrombopag, or 3’-{(2*Z*)-2-[1-(3,4-dimethylphenyl)-3-methyl-5-oxo-1,5-dihydro-4*H*-pyrazol-4-yliden] hydrazino}-2’ hydroxy-3- biphenylcarboxylic acid (CAS no. 496775-61-2), is a drug jointly discovered in 2006 by GlaxoSmithKline and Ligand Pharmaceuticals [1] that has been used, since its U.S. Food and Drug Administration (FDA)approval in 2008 [2], against thrombocytopenia, which is paucity of platelets in blood (thrombocytes). The conventional sketch of the molecular connectivity is shown in Scheme 1.

Eltrombopag is nowadays marketed in the USA under the tradename of Promacta and, in the EU, as Revolade (in both cases, by Novartis). More local brand names include Elbonix, Treptora, ETP, Eltopag, and Cytopag (mostly in southern Asiatic countries). The most common formulation is the bisolamine salt, which was found to be easy to produce, environmentally stable, and easily ingestible from oral suspensions [3]. Thus, the free acid is not commercialized per se, but is the starting material for the obtention of the marketed active pharmaceutical ingredient (API) that is obtained after precipitation from a methanolic solution with excess 2-aminoethanol [4].

APIs often exist in different solid-state forms, either crystalline or amorphous, and may be isolated as pure systems, or in hydrates, solvates, salts, and cocrystals [5,6]. Each solid form, including polymorphic ones, possesses its own molecular arrangement, dictated by distinct intermolecular interactions and conformationally driven crystal packing, leading to remarkably different physico-chemical properties. Consequently, melting points, crystal polarity and chirality, solubility, thermal and chemical stability (or inertness), crystal size and size distribution may show distinct features; these differences are further augmented by other effects typical of anisotropic media, such as shape of the crystals, preferential cleavage, hardness, compressibility and flowability [7,8,9]. These effects are particularly relevant during synthesis, isolation, and purification processes, as well as in the formulation steps (where rheological parameters become important during drug transport and tableting). Hygroscopicity and particle size and shape distributions may also affect shelf life of the marketed drug, and, more importantly, also pharmacokinetic and bioavailability properties may be dramatically changed, making the drug useless or even toxic [10].

As with many other cases in the vast literature on solid state forms of API, Eltrombopag also manifests the presence of several truly polymorphic (unsolvated) crystal phases, and, depending on the type of solvent from which is it recovered, also manifests many solvated phases containing different stoichiometric quantities of the solvent molecules in the crystal lattice [11,12,13]. To assist the reader in interpreting the large number of results obtained on this series of polymorphs and solvates, we have provided the correct assignments of the different species (or mixture thereof) at the end of this paper, clearing, to some extent, inexact or contradicting observations from the literature.

As Eltrombopag (free acid) is not the final, commonly supplied form to the patients, some of these issues, which remain valid for the Eltrombopag bisolamine system, vanish; however, knowledge of the crystal chemistry and the thermal stability and evolution paths of the many forms claimed in the patent literature (collectively listed in Table 1) is a must, as well as understanding the possible occurrence of polyphasic mixtures, (mis)taken, in the absence of a complete (at least metrical) characterization, for a single form. 

This paper, therefore, revises the zoo of Eltrombopag free acid polymorphs and solvates; tries to determine phase purity by conventional powder pattern indexing; it also provides, through variable-temperature in situ X-ray powder diffraction (XRPD) experiments, clues on the thermal stability and thermally induced evolution/desolvation of the Eltrombopag crystal phases which, not being isolated in milligram quantities within a Differential Scanning Calorimetry (DSC)apparatus, can be ideally prepared within pilot or industrial plants. More importantly, the complete list of the certified peak positions and relative intensities, obtained from crystallographic analysis, can be safely used for identifying a specific crystal phase, also if present in very minor quantities (say, below 1 wt % level), if accurate XRPD measurements are taken.

## 2. Results and Discussion

### 2.1. Preliminary Considerations

Form II was not reprepared, as it is not an Eltrombopag free acid (alone, or solvated) crystal phase, but, instead, a bis-ethanolamine salt, i.e., the marketed active pharmaceutical ingredient. Attempts to prepare Form III and Form IV, following the published procedures, did not result into the sought materials but, instead, lead to the isolation of Form XIV and Form I, respectively, later discussed. The unavailability of form IV impeded the preparation of Form VIII, which was derived by heating Form IV [11]. The preparation of Form XIII (in diethyl-ether) also failed and was attributed to the very scarce solubility of Form I, even at reflux conditions. Comparison of published data indicated that Form XV is a mixture of forms X and Xa (now prepared independently as pure crystal forms) and that Forms Z and H1 match the *N*,*N*-dimethylformamide (DMF) solvate form. The synthesis of Form XVI was not attempted, as it precipitated during the direct condensation of 3′-amino-2′-hydroxybiphenyl-3-carboxylix acid and 1-(3,4-dimethylphenyl)-3-methyl-1H-pyrazol-5-ol [11], not available in our laboratory. Finally, the amorphous material reported in [12], falling out of the scope of this study, was not considered.

**Figure 1:**
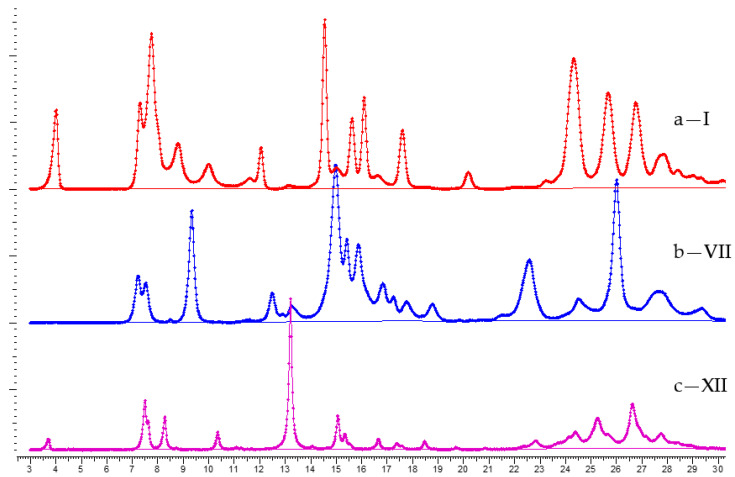
Raw X-ray powder diffraction (XRPD) data for unsolvated Eltrombopag free acid polymorphs ((**a**) Form I; (**b**) Form VII, and (**c**) Form XII), in the low-angle (3–30° 2θ) range. x and y axes: 2θ range, ° and intensities (a.u.), respectively. These raw data clearly demonstrate the higher crystallinity (narrower Bragg peaks) of Form XII (**c**) than for Form I (**a**) and Form VII (**b**).

**Figure 2:**
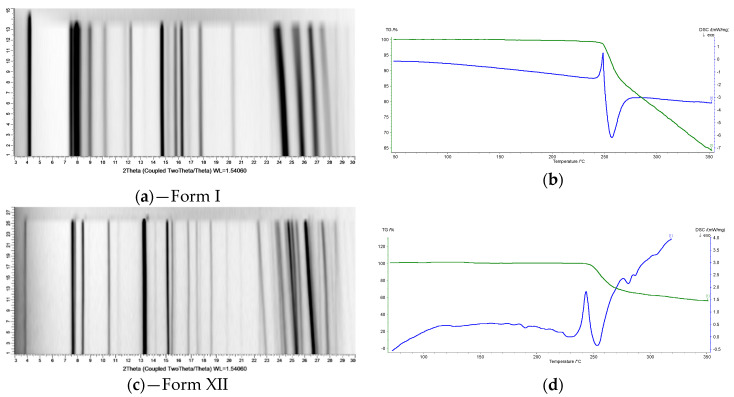
Variable Temperature X-ray Diffraction (VTXRD) traces of Form I (**a**) and Form XII (**c**). Horizontal scale: 2θ (°); vertical scale: progressive number of VTXRD measurements in isothermal conditions (in 20 °C steps each, in the 30–270 °C range). In panels (**b**) and (**d**), the pertinent TG (in green) and DSC (in blue) traces show that the unique thermally induced event, in both Form I and Form XII, is the incipient melting/decomposition occurring near 250 °C.

**Figure 3:**
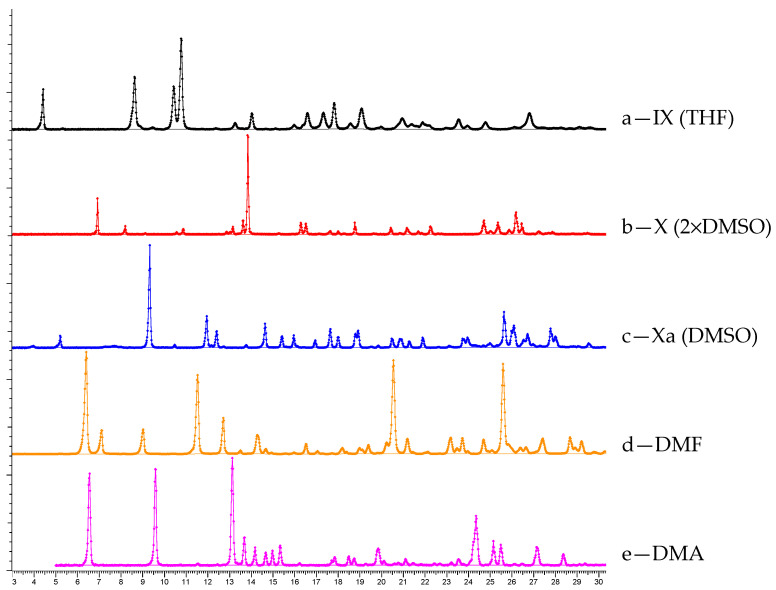
Raw XRPD data for solvated Eltrombopag free acid species: (**a**) Form IX; (**b**) Form X; (**c**) Form Xa; (**d**) DMF solvate; and (**e**) DMA solvate, in the low-angle (3–30° 2θ) range. x and y axes: 2θ range, ° and intensities (a.u.), respectively. These raw data clearly manifested the high crystallinity (narrow Bragg peaks) of all these stoichiometric solvates.

**Figure 4:**
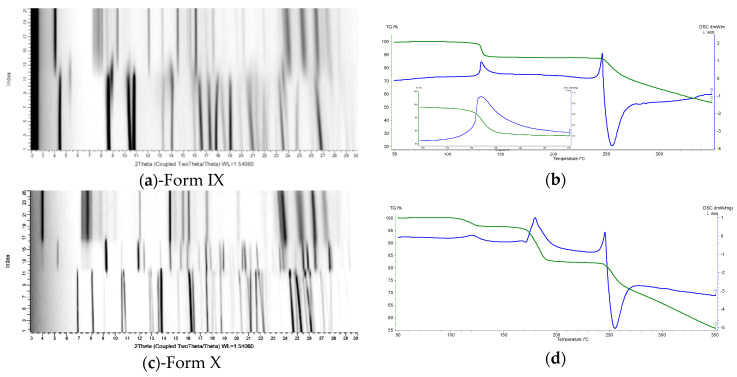

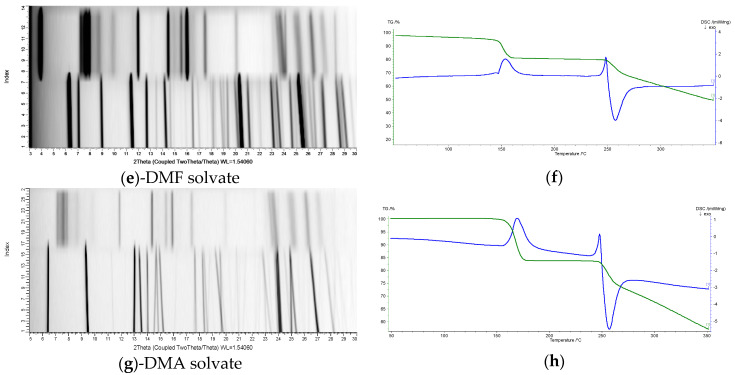
VTXRD of Form IX (**a**), Form X (**c**), DMF solvate (**e**), and DMA solvate (**g**). Horizontal scale: 2θ (°); vertical scale: progressive number of VTXRD measurement in isothermal conditions (in 20 °C steps each, in the 30–240 °C range). In panels (**b**,**d**,**f**,**h**), the pertinent TG (in green) and DSC (in blue) traces showing the thermally induced events, the intermediacy of the IXa and Xa phases discussed in the text, and the quantitative formation at high enough temperatures of Form I (decomposing near 250 °C). In panel (**b**), the inset shows the relevant high-T tail of the weak endotherm associated to the formation of Form IXa, discussed in the text.

**Figure 5:**
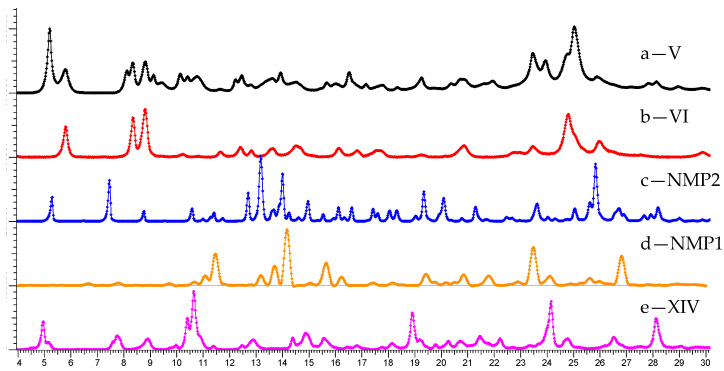
Raw XRPD data for solvated Eltrombopag free acid species: (**a**) Form V; (**b**) Form VI; (**c**) Form NMP (2:1) solvate; (**d**) NMP (1:1) solvate; and (**e**) Form XIV in the low-angle (4–30° 2θ) range. x and y axes: 2θ range, ° and intensities (a.u.), respectively. These raw data clearly manifested the high crystallinity (narrow Bragg peaks) of the NMP2 crystal phase, while broader peaks were observed for all other solvates.

**Figure 6:**
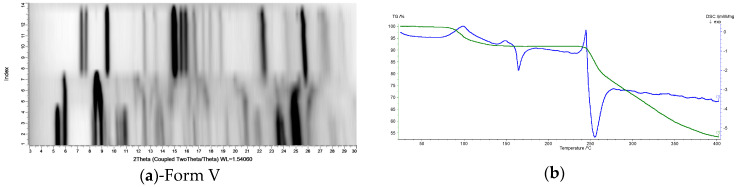

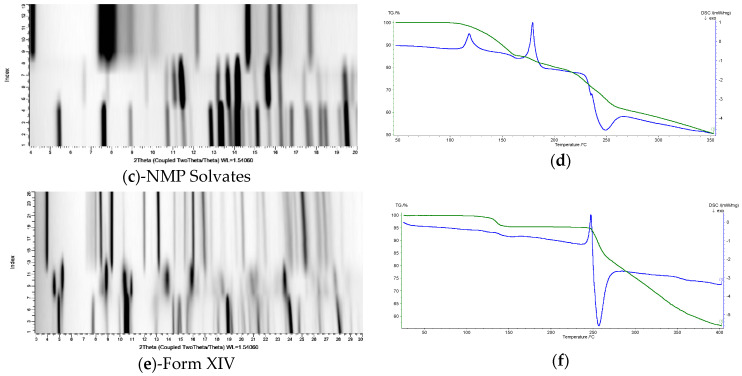
VTXRD and TG/DSC traces of Form V (**a**,**b**), the original NMP2-rich mixture of NMP1 and NMP2 crystal phases (**c**,**d**), and Form XIV (**e**,**f**). Horizontal scale: 2θ (°); vertical scale: progressive number of VTXRD measurements in isothermal conditions (in 20 °C steps each, typically in the 30–240 °C range).

All the crystal phases, whether unsolvated polymorphs or solvates of Etrombopag free acid, were characterized, in primis, by XRPD, which was used to assess phase purity and, after indexing, the unit cell size and shape, and ultimately their symmetry (crystal system and space group). As the raw data can be fruitfully used for fingerprinting, we propose in separate figures comparative plots of the unsolvated and solvated phases (Figure 1, Figure 2, Figure 3, Figure 4, Figure 5 and Figure 6); these figures contain the low-angle portions of the raw X-ray diffraction data, i.e., those that are normally used for identification purposes, as well for detecting the presence of contaminant phases. Table 2, Table 3, Table 4, Table 5, Table 6, Table 7, Table 8, Table 9, Table 10, Table 11, Table 12, Table 13 and Table 14 contain the observed 2θ angles, d-spacings, and relative intensities of the prominent indexed diffraction peaks (used in the cell determination procedure) and are reported within each pertinent paragraph. Comparative lists of our results and of the relevant crystal data, which include all determined unit cells, are shown in Table 15 and Table 16.

### 2.2. Preparation and Characterization of the Unsolvated Forms of Eltrombopag

Form I was easily reproduced by following the reported synthetic procedure [11]. As shown in Figure 1a, its diffraction peaks were relatively broad, and of markedly variable size and shape. Once cell refinement was performed, anisotropic particle sizes with average diameter near 50 nm were found. By applying slight modifications to the synthesis or thermal annealing, we could not improve this limited crystallinity. The fortunate isolation of a better crystallized material (ideally, Form XI), with the same powder diffraction pattern as Form I and sharper peaks, enabled the correct cell determination of a triclinic cell of a highly anisotropic shape. Looking at the proposed cell parameters, the classical dominant zone effect [14] was here present, weakening, to some (minor) extent, the accuracy of the evaluation of the very short real-space axis, falling slightly above 4 Å.

Form VII (Figure 1b) was prepared while heating, in the diffractometer cradle, powders of a mixture predominantly attributed to Form V (a tetrahydrofurane, THF, solvate); polyphasic in nature at room temperature, this mixture progressively evolved to the unsolvated Form VII through the intermediacy of another crystal phase (Form VI, later discussed). Obtained by high temperature treatment (above 180 °C), this material, after being cooled at room temperature, showed relatively broad peaks (as Form I did), but with a more limited spread in widths (speaking for less anisotropic particles, of average size close to around 45 nm).

Moreover, the already known Form XII was isolated following the reported synthetic procedure [11]. As shown in Figure 1c, its diffraction peaks were much narrower than those observed for Forms I and VII. For Form XII, particle sizes near 80 nm were found. Variable Temperature X-ray Diffraction (VTXRD) analysis, which did not show any desolvation process (in agreement with Thermogravimetry and Differential Scanning Calorimetry, TG/DSC, data), see Figure 2), confirmed that Form XII is a truly polymorphic form of Eltrombopag Forms I and VII, with a nearly identical molar volume, and, consequently, similar crystal density. The similarity of the strength of the intermolecular interactions in all three unsolvated forms was further demonstrated by the incipient melting/decomposition temperatures falling in the narrow 246–249 °C range.

All these unsolvated polymorphs are indefinitely stable, and did not show thermally induced solid–solid phase transitions from one to the other, as if highly reconstructive (and not displacive) molecular movements were necessary to cause polymorphic conversions. This is corroborated both by VTXRD and DSC measurements, gathered in Figure 2 for Forms I and XII. These VTXRD plots also indicated the most intense peaks above 22° 2θ, possessing *h* ≠ 0 indices and related to the presence of a very short **a** axis (slightly above 4 Å in both cases), shift upon heating to remarkably lower angles (i.e., to higher d-spacings) than *0kl* peaks, in line with a plausibly shared molecular stacking in Forms I and XII, along which thermal expansion effects are predominant.

### 2.3. Preparation and Characterization of the Solvated Forms of Eltrombopag: The Stoichiometric Phases

The crystal forms discussed in this section, that is, Form IX (a THF solvate), Form X (a dimethylsulfoxide-DMSO-solvate), and the *N*,*N*’-dimethylformamide (DMF) and *N*,*N*’-dimethylacetamide (DMA) solvates (no Roman numerals being assigned to the latter), were prepared using the precipitation protocols illustrated in [11,12]. Highly crystalline phases have been recovered, the low-angle portions of their XRPD traces being shown in Figure 3. Table 5, Table 6, Table 7, Table 8 and Table 9 report the experimental peak positions and intensities and their assigned hkl indices.

Although distinct, Form IX and Form X show some similarity—beyond crystallizing in the same triclinic space group (*P-1*), upon heating, they transformed into (distinct) intermediate phases (IXa and Xa, respectively), well before converting into unsolvated Form I (see Figure 4a,c). This process is easily understood for Form X, which possessed a 2:1 DMSO/Eltrombopag molecular ratio; in this case, partial desolvation (onset at around 114 °C) led to a 1:1 (i.e., monosolvate) monoclinic structure, and only at 175 °C was Form I quantitatively formed. VTXRD and TG/DSC analyses clearly showed the nature and extent of these processes (see Figure 4b,d). Separately prepared by heating powders of Form X (ex situ, at 150 °C), the 1:1 DMSO solvate was found to be stable at room temperature and provided an easily indexable XRPD trace (Figure 3c) and unit cell metrics apparently unrelated to that of Form X.

More intriguing is the formation of Form IXa, forming as an elusive crystalline phase while Form IX converted into Form I (see the VTXRD plot in Figure 4a where faint grey traces appear midway along the T coordinate (the *y*-axis). TG/DSC analysis of Form IX (see Figure 4b) indicated that the weak endothermic event occurring near 140 °C (onset at 130.5 °C) had a double component, with two closely overlapping peaks. Consequently, a XRPD trace of pure Form IXa could not be measured, nor were peak indexing and cell determination possible. From these data only, we could not assess the stoichiometry of this intangible phase, but, observing that the endothermic peak (DSC data) in the inset of Figure 4b appeared to be slightly retarded with respect to the weight loss (TG data), we put forward the hypothesis that IXa is an unsolvated (and unstable) crystalline polymorph of Forms I, VII, and XII, easily transforming into Form I upon heating.

Of much simple interpretation were the VTXRD and DSC data obtained on the highly crystalline solvated phase labelled DMF and DMA solvates, both of 1:1 solvent/Eltrombopag stoichiometry. Upon heating, they uniquely showed solvent desolvation (onsets at 148 and 161 °C, respectively), leading to Form I in a quantitative manner.

### 2.4. Preparation and Characterization of the Solvated Forms of Eltrombopag: Crystal Phases of Uncertain Stoichiometry and/or Complex Characterization

During the study of the crystal forms discussed in this section, and specifically Form V, Form XIV, and the *N*-methylpyrrolidone (NMP) solvate, we encountered unexpected difficulties, mostly related to the occurrence of polyphasic mixtures, some of which, upon heating, generated much more understandable XRD traces, attributed to rigorously monophasic materials.

Form V, prepared following the literature recipe [11], was indeed found to contain a polyphasic mixture, with a non-indexable XRPD pattern, as shown in Figure 5a. As demonstrated by the VTXRD plot illustrated in Figure 6a, it can be transformed, above 90 °C, into a pure crystal phase, that is, Form VI, the cell symmetry and metrics of which are collected in Table 16. Its low-angle raw XRPD data were also inserted in Figure 5. As anticipated, further heating above 160 °C promoted the formation of the unsolvated phase Form VII, which could not be precipitated from solution nor obtained by thermally induced transformation of any of the other Eltrombopag crystal forms characterized in this work.

Following the published protocol [12], the so-called NMP solvate was also not easily isolated. The crude product recovered after filtration, indeed, was found to be a polyphasic mixture, dominated by an NMP-rich crystal phase (see the XRPD trace in Figure 5c). Selective peak choices enabled the determination of the monoclinic unit cell, with a volume corresponding to a 2:1 NMP/Eltrombopag molar ratio. Accordingly, we labeled this form NMP2, while that obtained after heating it above 110 °C was found to be a crystallographically pure material of 1:1 NMP/Eltrombopag ratio, herein identified as NMP1 (see Figure 5d). Interestingly, the XRPD data of NMP1 phase perfectly matched the NMP solvate phase originally characterized in [12]. Heating Form NMP1 above 180 °C generates Form I, as most (but not all) solvated crystal forms do.

Moreover, the XRPD trace of form XIV and its evolution with temperature (Figure 5e and Figure 6e, respectively) proved to be a challenging task for their complete interpretation. At room temperature, an ethylacetate (EtOAc) solvate of triclinic symmetry was identified, with crystal data inserted in Table 16. Near 90 °C, new peaks appeared, and the formation of a new phase, labeled as XIVa, was observed. The poor stability of this phase and the broad feature of its Bragg peaks prevented further metrical characterization. Above 120 °C, Form I was obtained, but, unexpectedly, a few sharp peaks of an additional and unknown crystal phase (here called XIVb, not indexable, as the number of independent reflections not overlapped with those of the predominant Form I was too low) were detected.

### 2.5. Comparative Crystal Chemistry

Eltrombopag free acid and its solvated forms crystallize in low symmetry crystal systems, mostly triclinic, and only in two cases as primitive monoclinic cells (Forms Xa and NMP1 solvate). In all cases, assuming that centrosymmetric space groups were present, Eltrombopag (pseudo)polymorphs showed a Z = 1 value, with Z being the number of formula units in the asymmetric portion of the unit cell. Moreover, thanks to this fortunate occurrence, peak indexing and cell determination proved to be a soluble task, even in the presence of broadened peaks or when only polyphasic samples were available.

**Figure 7:**
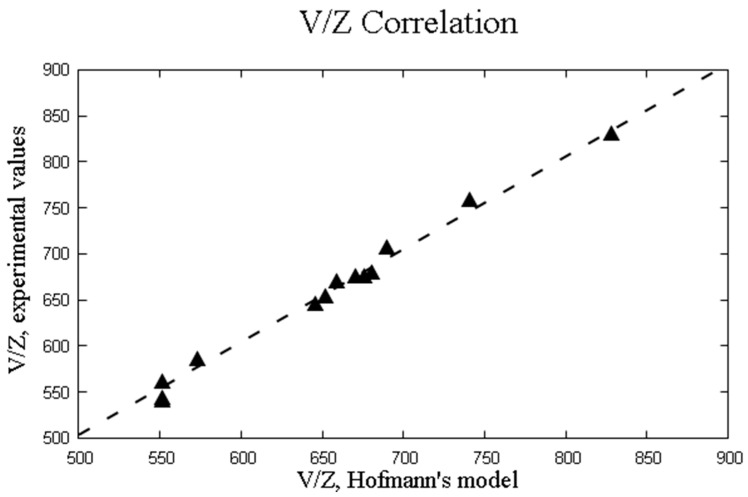
Molar volume correlation (values in Å^3^) for the different crystal phases indexed in this paper, showing how good quality XRPD data may be uses in a predictive manner to assess the exact solvent nature and stoichiometry within the large class of Eltrombopag solvates. The dashed line refers to the linear fit defined by *y* = 1.007*x*.

While thermal methods (above all, TG) may properly suggest the nature, and extent, of the solvation process, the synthetic approaches we used strongly suggest which molecules are likely to be present. A further, very compelling set of observations (the molar volumes, that is, the V_cell_/Z values, included in Table 16 in Å^3^/Z form, and easily transformed into V_m_ in milliliters by using the N_Av_/10^24^ conversion factor) also provided indirect measures of the crystal phase stoichiometries. Grounded on the well-known additivity of atomic volumes, valid in the absence of permanent porosity and crystal voids, Hofmann derived, from a statistical analysis of crystallographic databases, effective size effects for all atoms crystallizing in organics and in metallorganic compounds. [15] Accordingly, the experimentally derived molar volumes inserted in Table 16 were compared with those calculated using Hofmann’s Table [15], under the constraint that the crystal phase stoichiometry was known. The high correlation among the two sets is depicted in Figure 7, where, if the correct estimate of the type and number of occluded solvent molecules is correct, a linear regression with a null intercept should provide a slope close to unity. Data shown in Figure 7 were linearly fit by the y = mx straight line, resulting in m = 1.007(4), with an *R^2^* value of 0.9998. Notably, had wrong chemical formulae been inserted, such an excellent fit would not have been reached. This is particularly diriment in the case of Form V (a mixed solvent phase), of the monohydrated Form VI derived therefrom, and of the 2:1 solvent/Eltrombopag crystal phases (solvent = DMSO or NMP) with different, although still stoichiometric, solvent content.

## 3. Materials and Methods

### 3.1. Materials

A 50 g industrial batch of Eltrombopag free acid was supplied by Chemessentia and characterized by solution ^1^H-NMR and ^13^C-NMR spectroscopy (NMR Avance 400 spectrometer, Bruker, Billerica, MA, USA), as well as by XRPD. The crude product was found to consist of a physical mixture of Form I, Form V, and Form VI. Accordingly, ^1^H-NMR showed additional peaks of the residual solvent (THF–water). ^1^H-NMR (400 MHz, DMSO-d_6_, 25 °C): δ 13.78 (bs, 1H), 13.04 (bs, 1H), 9.71 (bs, 1H), 8.17 (s, 1H), 7.99 (d, *J* = 7.6 Hz, 1H), 7.84 (d, *J* = 7.6 Hz, 1H), 7.75 (s, 2H), 7.72–7.63 (m, 2H), 7.30–7.15 (m, 3H), 2.37 (s, 3H), 2.31 (s, 3H), 2.26 (s, 3H). ^13^C-NMR (100 MHz, DMSO-d_6_, 25 °C): δ 167.22, 156.86, 147.78, 142.52, 137.65, 136.77, 135.76, 133.35, 132.78, 132.19, 131.04, 130.61, 129.88, 129.76, 128.89, 128.32, 128.26, 127.09, 121.93, 118.88, 115.32, 113.95, 19.63, 18.82, 11.52.

Solvents (acetic acid (Sigma Aldrich, 99%), acetone (Emplura, 99%), anisole (Sigma Aldrich, 99%), DMA (Merck, >98%), DMF (Sigma Aldrich, 99.8%), DMSO (Emplura, >98%), ethanol (Emsure, 99%), ethyl acetate (Carlo Erba, >99.8%), methanol (Sigma Aldrich, 99.8%), NMP (Sigma Aldrich, 99%), THF (Sigma Aldrich, 99.9%)) were used without purification.

### 3.2. Preparation of the Different Eltrombopag Crystal Phases

When, in the following sections, an unspecified form of Eltrombopag was used as a starting material, we refer to the crude product (the as-received laboratory batch described above in Section 2.1, consisting of a polyphasic mixture).

#### 3.2.1. Preparation of Eltrombopag Form I (Unsolvated)

A total of 2.50 g of Eltrombopag was dissolved in 260 mL of glacial acetic acid (≥99%) in a four-neck 500 mL round bottom flask equipped with condenser and heated through an oil bath at the refluxing temperature of glacial acetic acid (118 °C). Once a solution was obtained, the hot mixture was filtered on a porous septum using a water vacuum pump and left to crystallize overnight. The obtained product was filtered, collected, and dried at 35 °C under vacuum. We obtained 1.627 g of a bright orange product.

#### 3.2.2. Preparation of Eltrombopag Form VII (Unsolvated)

Eltrombopag Form V (200 mg) was deposited on a Petri dish and heated in an oven, initially at 110 °C for 4 h and then at 160 °C for 2 h. The quantitatively recovered powder, after loss of the solvent, showed a significantly different diffraction trace than Form I and Form XII, witnessing the formation of a new unsolvated crystal phase.

#### 3.2.3. Preparation of Eltrombopag Form XI (Unsolvated)

A total of 200 mg of Eltrombopag was put in a 250 mL two-neck round bottom flask equipped with condenser, and 60 mL of acetone was added. The suspension was heated at the refluxing temperature until a clear solution was formed. The hot mixture was then filtered on a porous septum and the filtered solution was left at room temperature until complete evaporation of the solvent. Yield, 90 mg. XRPD clearly showed the identity of Form XI and Form I patterns, thus dismissing Form XI as being an independent crystal phase.

#### 3.2.4. Preparation of Eltrombopag Form XII (Unsolvated)

Eltrombopag Form I (20 mg) was dissolved in anisole (6 mL) using a two-neck 25 mL round bottom flask with heating and stirring until complete dissolution was achieved. The solution was then left at room temperature for 3 days. Agglomerated spherulites formed, filtered-off with the aid of gentle vacuum (yield: 12 mg).

#### 3.2.5. Preparation of Eltrombopag Form IX (THF Solvate)

Eltrombopag Form I (20 mg) was dissolved in THF (2 mL) using a 3 mL cylindrical test flask with moderate heating and shaking. The mixture was then cooled and left at room temperature until complete evaporation of the solvent, leading to 20 mg of an orange powder.

#### 3.2.6. Preparation of Eltrombopag Form X (2:1: DMSO/Eltrombopag Solvate)

Eltrombopag Form I (20 mg) was dissolved in DMSO (2 mL) using a 3 mL cylindrical test flask with moderate heating and shaking. The mixture was then cooled and left at room temperature until a shiny orange powder was formed, later collected by filtration (12 mg).

#### 3.2.7. Preparation of Eltrombopag Form Xa (1:1 DMSO Solvate)

The new Eltrombopag Form Xa was detected during a thermodiffractometric analysis of Form X. Indeed, heating Form X at around 140 °C quantitatively generates a still unknown crystal phase (Form Xa, in the following). Form Xa, stable after cooling down to room temperature, was then easily recovered and analyzed.

#### 3.2.8. Preparation of Eltrombopag DMF Solvate

A total of 300 mg of Eltrombopag and 1.05 mL of *N*,*N*-dimethylformamide were placed in a two-neck round bottom flask (10 mL) equipped with a condenser, and the suspension was heated via an oil bath at 70 °C under vigorous stirring for 30 min. Then, 1.05 mL of absolute ethanol was added, and the mixture was stirred for 30 additional min, maintaining the working temperature constant (70 °C); during this time, the formation of a solid precipitate was observed. The mixture was then cooled to room temperature and kept under stirring for additional 15 h. The obtained solid was filtered, washed with absolute ethanol, and dried in air, leading to the recovery of 266 mg of a light orange powder.

#### 3.2.9. Preparation of Eltrombopag DMA Solvate

A total of 200 mg of Eltrombopag and 0.7 mL of *N*,*N*-dimethylacetamide were placed in a two-neck round bottom flask (10 mL) equipped with a condenser and heated via an oil bath until a clear solution at 80 °C formed. The mixture was stirred at this temperature for 30 min. Subsequently, 0.7 mL of absolute ethanol was added and the mixture was stirred for 30 additional min, maintaining the working temperature constant (80 °C); during this time, the formation of a solid precipitate was observed. The mixture was then cooled to room temperature and kept under stirring for 1 additional hour. The obtained solid was filtered, washed with 5 mL of absolute ethanol, and dried in air, leading to the recovery of 120 mg of a bright orange powder.

#### 3.2.10. Preparation of Eltrombopag NMP (NMP2 and NMP1) Solvates

A total of 300 mg of Eltrombopag and 1.2 mL of NMP were placed in a two-neck round bottom flask (10 mL) equipped with condenser and heated under vigorous stirring for about 30 min until a clear solution at 75 °C was formed. Then, 1.8 mL of ethanol was added and the mixture was stirred for additional 30 min, maintaining the working temperature constant at 75 °C. The mixture was then cooled to room temperature and stirred for 1 additional hour. The obtained precipitate was filtered, washed with 0.5 mL of ethanol, and dried under vacuum (yield: 295 mg), and then characterized as the NMP2 crystal phase, containing NMP/Eltrombopag in a 2:1 molar ratio. Heating of this phase at 130 °C during VTXRD measurements promoted the quantitative formation of a different NMP solvate, labeled as NMP1, with a 1:1 stoichiometric ratio.

#### 3.2.11. Preparation of Eltrombopag Form V

To 3.0 g of Eltrombopag placed in a 100 mL two-neck round bottom flask equipped with condenser, we added 17 mL of THF. The mixture was heated at the refluxing temperature (90 °C) until a clear solution was obtained. Then, water (17 mL) was added dropwise at this temperature and the solution was later cooled to room temperature and left under vigorous stirring for 1 additional hour. The precipitate was filtered, washed with water, and dried for 2 h at 50 °C. The product was freeze-dried at 0.05 mbar and −53 °C in order to remove the residual intergrain (non-structural) water molecules.

#### 3.2.12. Preparation of Eltrombopag Form VI

Eltrombopag Form V (200 mg) was placed in an alumina crucible and heated, with a heating ramp rate of 10 °C min^−1^, in a muffle furnace up to 110 °C and kept at this temperature for 20 min before cooling to room temperature. Quantitative transformation from form V to form VI was observed (189 mg of material were recovered, in line with a possible monohydrate formulation of Form VI).

### 3.3. X-ray Powder Diffraction Characterization

#### 3.3.1. Experimental Conditions

All powdered samples were gently ground in an agate mortar and deposited in the hollow of a zero-background plate (a 0.2 mm deep insert in a mis-cut silicon monocrystal). Diffraction data were collected at room temperature using a Bruker D8 AXS Advance diffractometer, operating in θ:θ mode, and equipped with a 1D position sensitive detector (Lynxeye). Ni-filtered Cu-Kα_1,2_ radiation; generator setting: 40 kV, 40 mA; goniometer radius = 300 mm; 2.5° Soller slits in the primary beam, divergence slit = 0.5°. A custom-made antiscatter steel knife was positioned about 2.0 mm above the sample to limit the presence of parasitic X-rays entering the detector window. Data were acquired in the full 3–105° 2θ range, in step scan mode: ∆2θ = 0.02°, counting time 15 s, overall duration: around 21 h.

#### 3.3.2. Variable Temperature X-ray Diffraction

These experiments were performed on the same equipment used in the previous section, modified by the presence of a custom-made sample heater (provided by Officine Elettrotecniche di Tenno, Ponte Arche, Italy), operated by an external controller with a nominal thermal stability of ±0.1 °C. As the thermocouple is at the bottom of the aluminum sample holder and the surface of the sample is in contact with the air, some thermal gradients are expected, with the set temperature being a few degrees higher than the average one. DSC temperatures (discussed below) are then much more reliable than the XRPD ones.

#### 3.3.3. Determination of Crystallographic Unit Cell and Space Group Symmetry

Standard peak search, followed by profile fitting and the fundamental parameters approach [16], provided a starting list of around 20 well defined, low-angle peak positions, fed to the indexing routine of TOPAS-R [17]. Through the singular value decomposition approach [18], cell determination and reflection hkl indices were derived, together with the goodness-of-fit parameters, GOF [19], listed at the bottom of Table 16. Allowance for sample-displacement, transparency, and flat-plate aberration errors was given, in the form of a constant zero-error shift, valid for a small low-angle portion of the data. Space group symmetry was assessed, coupling density considerations and, where pertinent, the analysis of systematic absences.

### 3.4. Thermal Analyses

Simultaneous thermogravimetric and differential scanning calorimetric analyses were performed under nitrogen atmosphere, in the RT to 400 °C range on a STA 409 PC Luxx equipment (Netzsch, Selb, Germany), using sample weights in the 5–10 mg range and open alumina crucibles at a 10 °C min^−1^ scan rate. DSC onset points and TG mass losses were determined by Proteus Software version 4.8.1 (Netzsch, Selb, Germany).

## 4. Conclusions

In this paper, we reported the controlled syntheses of Eltrombopag free acid in its pure form or in a number of distinct crystalline polymorphs and solvates. The complete cell determination and peak indexing processes from accurate X-ray powder diffraction measurements certified the absence of contaminants and made the proposed crystallographic features a sound basis for polymoph/impurity detection in a large number of solid forms of Eltrombopag obtained under different operational conditions. The stability of the different (pseudo)polymorphs upon heating and their desolvation paths were studied, leading to the discovery of new intermediate phases or even of unexpected (room temperature-stable) forms of the pristine unsolvated material (Form I). Work can be anticipated in the direction of determining the full crystal structures by structural laboratory powder diffraction methods, very much in the way recently performed by us (and others) on crystal phases of drugs with molecules of moderate complexity [20]. Indeed, although promising structureless methods for polymorph quantification have appeared [21], this additional valuable information may help in quantifying contaminants and even amorphous phases in complex mixtures once the complete structural models are available [22].

## Figures and Tables

**Table 1:** Known Eltrombopag free acid polymorphs and solvates described in the literature.

Form Name	Nature	Reference	Comments
I	Crystalline, unsolvated	[11]	Reprepared
II	Crystalline, bis-ethanolamine salt	[11]	Not relevant (salt)
III	Crystalline, hydrate	[11]	Form XIV obtained
IV	Crystalline, not specified	[11]	Form I obtained
V	Crystalline, THF/water solvate	[11]	Reprepared
VI	Crystalline, not specified	[11]	Reprepared
VII	Crystalline, not specified	[11]	Reprepared
VIII	Crystalline, not specified	[11]	Not obtained
IX	Crystalline, not specified	[11]	Reprepared
X	Crystalline, not specified	[11]	Reprepared
XI	Crystalline, not specified	[11]	Reprepared
XII	Crystalline, not specified	[11]	Reprepared
XIII	Crystalline, not specified	[11]	Not obtained
XIV	Crystalline, not specified	[11]	Reprepared
XV	Crystalline, not specified	[11]	Not attempted (mixture)
XVI	Crystalline, monohydrate	[11]	Synthesis not attempted
Z	Crystalline, not specified	[12]	Equal to DMF solvate
Amorphous	Fully amorphous	[12]	Not relevant (amorphous)
DMF solvate	Crystalline, DMF solvate	[12]	Reprepared
DMA solvate	Crystalline, DMA solvate	[12]	Reprepared
NMP solvate	Crystalline, NMP solvate	[12]	Reprepared
H1	Crystalline, not specified	[13]	Equal to DMF solvate

**Table 2:** List of observed 2θ angles, d-spacings, and relative intensities, jointly with reflection indices, for Eltrombopag Form I.

2θ Angle, °	d-Spacing, Å	I_rel_	h k l
3.999	22.074	50	0 0 1
7.278	12.135	52	0 1 0
7.761	11.380	93	0 −1 1
7.989	11.057	37	0 0 2
8.794	10.046	28	0 1 1
10.003	8.834	15	0 −1 2
11.613	7.613	6	0 1 2
12.034	7.348	25	0 0 3
13.135	6.734	2	0 −1 3
14.538	6.087	100	0 2 0
14.997	5.902	23	0 1 3
15.627	5.665	42	0 2 1
16.086	5.505	55	0 0 4
16.673	5.312	8	0 −1 4
17.605	5.033	35	0 2 2
18.626	4.760	1	0 1 4
20.166	4.399	10	0 0 5
21.418	4.145	<1	1 0 0
21.922	4.051	1	1 0 −1
22.512	3.946	1	1 1 0

**Table 3:** List of observed 2θ angles, d-spacings, and relative intensities, jointly with reflection indices, for Eltrombopag Form VII.

2θ Angle, °	d-Spacing, Å	I_rel_	h k l
7.243	12.193	28	0 0 1
7.530	11.729	24	0 1 0
9.343	9.457	67	0 −1 1
11.520	7.675	2	0 1 1
12.501	7.074	17	1 0 −1
12.878	6.868	6	1 −1 0
13.261	6.671	10	1 −1 −1
14.570	6.074	16	0 0 2
14.982	5.908	100	0 −1 2
15.425	5.739	50	0 −2 1
15.880	5.576	46	1 1 −1
16.825	5.265	23	1 0 1
17.256	5.134	16	1 −1 −2
17.770	4.987	13	1 −2 −1
18.787	4.719	11	0 −2 2
21.442	4.140	4	1 −2 −2
22.566	3.936	38	1 −3 0
24.073	3.693	5	0 −2 3
24.516	3.628	14	2 0 −1
24.827	3.583	10	2 −1 −2

**Table 4:** List of observed 2θ angles, d-spacings, and relative intensities, jointly with reflection indices, for Eltrombopag Form XII.

2θ Angle, °	d-Spacing, Å	I_rel_	h k l
3.722	23.715	7	0 0 1
7.513	11.757	33	0 0 2
7.631	11.575	19	0 1 0
8.294	10.650	22	0 1 1
8.740	10.109	1	0 −1 1
10.374	8.520	11	0 1 2
11.100	7.964	2	0 −1 2
11.280	7.838	1	0 0 3
13.226	6.688	100	0 1 3
14.109	6.271	2	0 −1 3
15.075	5.872	22	0 0 4
15.358	5.764	10	0 2 0
15.544	5.695	4	0 2 1
16.641	5.322	7	0 2 2
17.386	5.096	4	0 −1 4
17.552	5.048	3	0 −2 2
18.490	4.794	5	0 2 3
18.904	4.690	<1	0 0 5
19.704	4.501	2	0 −2 3
20.877	4.251	1	0 2 4
22.346	3.975	2	0 −2 4
22.829	3.892	6	0 0 6
23.720	3.748	4	0 2 5
24.105	3.688	8	1 0 1
24.388	3.646	12	1 −1 0
25.260	3.522	21	1 1 −4
25.664	3.468	10	1 0 −5
26.623	3.345	31	0 0 7
26.906	3.311	14	1 2 −2
27.182	3.278	9	1 2 0
27.760	3.211	11	1 0 −6

**Table 5:** List of observed 2θ angles, d-spacings, and relative intensities, jointly with reflection indices, for Eltrombopag Form IX.

2θ Angle, °	d-Spacing, Å	I_rel_	h k l
4.415	19.997	42	0 0 1
8.481	10.417	55	0 1 0
8.625	10.243	4	0 −1 1
10.438	8.468	44	0 1 1
10.756	8.217	100	0 −1 2
13.256	6.673	6	0 0 3
14.011	6.315	16	0 −1 3
14.677	6.030	<1	1 0 −1
15.101	5.861	<1	1 0 1
15.968	5.545	4	1 −1 1
16.370	5.410	4	1 0 −2
16.581	5.342	17	0 −2 1
17.306	5.119	17	1 1 −1
17.800	4.978	27	0 −1 4
18.571	4.773	6	0 2 1
19.059	4.652	22	0 −2 3
19.817	4.476	1	1 −1 3
20.002	4.435	2	1 0 3
20.769	4.273	5	1 1 2
20.970	4.232	12	0 2 2

**Table 6:** List of observed 2θ angles, d-spacings, and relative intensities, jointly with reflection indices, for Eltrombopag Form X.

2θ Angle, °	d-Spacing, Å	I_rel_	h k l
6.927	12.749	36	0 0 1
8.210	10.760	8	0 1 0
8.740	10.109	<1	1 0 1
9.135	9.672	1	1 0 0
10.571	8.361	2	0 1 1
10.878	8.126	5	0 −1 1
12.870	6.872	2	1 0 2
13.035	6.786	2	1 −1 1
13.159	6.722	8	1 −1 0
13.631	6.491	14	1 0 −1
13.851	6.388	100	0 0 2
15.279	5.794	1	1 1 −1
15.920	5.562	<1	0 1 2
16.284	5.438	12	0 −1 2
16.522	5.360	11	1 −1 −1
17.164	5.162	<1	1 2 1
17.640	5.023	3	0 2 1
18.009	4.921	3	0 −2 1
18.282	4.848	1	2 0 0
18.759	4.726	12	1 0 3

**Table 7:** List of observed 2θ angles, d-spacings, and relative intensities, jointly with reflection indices, for Eltrombopag Form Xa.

2θ Angle, °	d-Spacing, Å	I_rel_	h k l
5.212	16.941	11	0 0 1
9.338	9.462	100	2 0 0
10.482	8.432	2	0 0 2
11.910	7.424	15	2 0 −1
11.954	7.397	28	2 0 2
12.166	7.269	2	1 1 0
12.411	7.125	15	0 1 1
12.737	6.944	1	1 1 1
13.778	6.422	2	1 −1 −1
14.635	6.047	22	2 1 0
15.418	5.742	10	0 1 2
15.961	5.548	11	2 0 −2
16.944	5.228	7	1 −1 −2
17.642	5.023	17	3 1 1
18.007	4.921	10	4 0 1
18.808	4.714	12	4 0 2
18.928	4.684	15	1 1 3
19.522	4.543	1	2 −1 −2
19.840	4.471	2	3 −1 −1
20.488	4.331	8	2 0 −3

**Table 8:** List of observed 2θ angles, d-spacings, and relative intensities, jointly with reflection indices, for Eltrombopag DMF solvate.

2θ Angle, °	d-Spacing, Å	I_rel_	h k l
6.406	13.784	100	0 0 2
7.117	12.410	24	0 1 1
9.026	9.789	24	0 1 2
11.535	7.664	4	0 1 3
12.716	6.955	75	0 2 0
13.110	6.747	35	0 2 1
13.220	6.691	3	1 0 1
13.501	6.552	3	−1 0 1
14.278	6.197	18	0 2 2
14.332	6.174	15	0 1 4
14.687	6.026	5	1 1 1
14.944	5.923	1	−1 −1 1
15.590	5.679	<1	1 1 2
15.986	5.539	1	0 2 3
16.534	5.357	9	−1 0 3
17.056	5.194	3	1 1 3
17.300	5.121	<1	0 1 5
17.744	4.994	<1	−1 −1 3
18.206	4.868	6	1 2 0
18.388	4.820	2	1 2 1

**Table 9:** List of observed 2θ angles, d-spacings, and relative intensities, jointly with reflection indices, for Eltrombopag DMA solvate.

2θ Angle, °	d-Spacing, Å	I_rel_	h k l
6.562	13.457	85	0 0 1
9.578	9.226	89	0 1 1
11.530	7.668	1	1 0 0
12.410	7.126	<1	1 0 1
13.132	6.736	100	0 0 2
13.675	6.469	26	0 2 0
14.158	6.250	16	1 −1 1
14.646	6.043	11	0 −1 2
14.972	5.912	13	0 1 2
15.331	5.774	18	0 2 1
16.202	5.466	2	1 0 2
17.703	5.005	4	1 1 2
17.839	4.968	7	1 2 0
18.484	4.796	8	1 −2 1
18.722	4.735	6	1 0 −2
19.251	4.606	1	0 2 2
19.781	4.484	13	1 1 −2
19.861	4.466	15	1 −2 −1
20.107	4.412	4	1 −1 −2

**Table 10:** List of observed 2θ angles, d-spacings, and relative intensities, jointly with reflection indices, for Eltrombopag Form V.

2θ Angle, °	d-Spacing, Å	I_rel_	h k l
5.210	16.946	100	0 0 1
8.128	10.869	34	0 1 0
9.127	9.681	27	0 −1 1
10.142	8.713	29	0 1 1
10.417	8.485	25	0 0 2
12.238	7.226	20	1 0 0
12.464	7.095	26	0 −1 2
13.947	6.344	31	0 1 2
15.687	5.644	15	0 0 3
16.534	5.357	31	0 −2 1
17.162	5.162	13	1 −1 2
18.355	4.829	8	0 −2 2
19.266	4.603	23	1 −2 1
19.750	4.491	7	1 −2 −1
20.378	4.354	13	0 2 2
21.627	4.105	14	0 −1 4
21.942	4.047	18	1 2 0
23.465	3.788	62	1 0 −4
23.948	3.712	50	0 2 3
24.771	3.591	60	2 0 −1
25.037	3.553	100	2 −1 −1

**Table 11:** List of observed 2θ angles, d-spacings, and relative intensities, jointly with reflection indices, for Eltrombopag Form VI.

2θ Angle, °	d-Spacing, Å	I_rel_	h k l
5.815	15.183	62	0 0 1
8.361	10.566	80	0 1 0
8.820	10.016	100	0 1 1
11.659	7.583	11	0 0 2
12.418	7.121	20	0 1 2
12.837	6.890	13	1 0 0
13.560	6.524	15	1 0 −1
13.666	6.474	17	1 1 0
14.493	6.106	22	1 1 1
14.673	6.032	19	1 0 1
16.136	5.488	18	0 −1 2
16.848	5.257	13	0 2 0
17.376	5.099	8	1 1 2
17.525	5.056	13	0 0 3
17.740	4.995	13	0 2 2
18.750	4.728	1	1 2 0
19.252	4.606	4	0 −2 1
20.859	4.255	23	0 2 3
22.736	3.907	10	1 −2 −1

**Table 12:** List of observed 2θ angles, d-spacings, and relative intensities, jointly with reflection indices, for Eltrombopag NMP2.

2θ Angle, °	d-Spacing, Å	I_rel_	h k l
5.284	16.708	37	0 0 2
7.463	11.835	63	0 1 1
8.764	10.080	15	0 1 2
10.590	8.346	19	0 1 3
11.738	7.532	4	1 0 2
12.705	6.961	44	0 1 4
13.175	6.714	100	−1 0 2
14.251	6.209	12	0 2 1
14.621	6.053	6	1 1 3
14.965	5.915	31	−1 −1 2
15.940	5.555	4	0 0 6
16.120	5.493	21	0 2 3
16.619	5.330	21	−1 0 4
17.423	5.085	18	0 1 6
17.604	5.033	12	0 2 4
18.047	4.911	16	1 2 0
18.318	4.839	16	1 2 2
19.041	4.657	8	1 2 3
19.348	4.583	46	0 2 5
19.970	4.442	11	0 0 2

**Table 13:** List of observed 2θ angles, d-spacings, and relative intensities, jointly with reflection indices, for Eltrombopag NMP1.

2θ Angle, °	d-Spacing, Å	I_rel_	h k l
6.525	13.534	3	0 0 1
7.656	11.536	4	0 1 0
9.589	9.216	3	0 −1 1
10.542	8.384	1	0 1 1
10.941	8.079	6	1 −1 0
11.306	7.819	18	1 −1 1
11.606	7.618	53	1 0 1
13.057	6.774	17	0 0 2
13.561	6.523	36	1 0 −1
14.030	6.307	100	1 −1 −1
14.888	5.945	<1	1 −1 2
15.503	5.710	37	1 −2 0
16.081	5.507	16	0 −2 1
17.274	5.129	4	0 2 1
17.987	4.927	<1	1 −2 2
18.350	4.830	4	1 0 −2
19.266	4.603	16	1 1 2
19.610	4.523	6	0 0 3

**Table 14:** List of observed 2θ angles, d-spacings, and relative intensities, jointly with reflection indices, for Eltrombopag Form XIV.

2θ Angle, °	d-Spacing, Å	I_rel_	h k l
4.959	17.804	50	0 0 1
7.761	11.380	14	0 1 0
7.870	11.224	23	0 1 1
9.972	8.862	7	0 0 2
10.406	8.494	54	0 −1 1
10.650	8.299	100	0 1 2
14.386	6.151	20	0 −1 2
14.890	5.944	27	0 2 1
15.577	5.684	19	0 2 0
16.430	5.390	4	1 −1 2
16.826	5.264	7	1 −1 −1
17.763	4.989	4	0 −2 1
18.132	4.888	10	0 2 3
18.912	4.688	65	0 −1 3
19.208	4.617	16	1 −1 3
19.455	4.558	5	1 −2 1
19.784	4.483	8	1 −1 −2
20.279	4.375	14	1 0 4
20.640	4.299	12	1 0 −2
20.752	4.276	14	1 1 4
21.467	4.136	23	0 2 4
21.770	4.079	10	1 −2 −2

**Table 15:** Assignment of Eltrombopag free acid polymorphs and solvates characterized in this work, using, when possible, the original labeling, with the inclusion of the newly discovered features.

Form	Solvent	Unit Cell	Comments on Original Data	Ref.
I	None	Indexed		[11]
V	THF/H_2_O		Mixture of THF/H_2_O solvates	[11]
VI	H_2_O	Indexed		[11]
VII	None	Indexed		[11]
IX	THF	Indexed		[11]
X	2 × DMSO	Indexed		[11]
Xa	1 × DMSO	Indexed		[11]
XI	None		Mixture rich in Form I	[11]
XII	None	Indexed		[11]
XIV	EtOAc	Indexed		[11]
XV	DMSO		Mixture rich in Form 1x DMSO	[11]
Z	DMF		= DMF solvate	[12]
DMF solvate	DMF	Indexed		[12]
DMA solvate	DMA	Indexed		[12]
NMP2 solvate	2 × NMP	Indexed		[12]
NMP1 solvate	1 × NMP	Indexed		[12]
H1	DMF		= DMF solvate	[13]

**Table 16:** Synoptic collection of crystal data for the different phases of Eltrombopag.

Form	I	V	VI	VII	IX	X	Xa	XII	XIV	DMF	DMA	NMP2	NMP1
Solvent	none	THF, H_2_O	H_2_O	none	THF	2 × DMSO	1 × DMSO	none	EtOAc	DMF	DMA	2 × NMP	1 × NMP
Fw, g mol^−1^	442.51	532.65	460.53	442.51	514.63	598.81	520.66	442.51	530.63	515.62	529.65	640.81	541.66
System	Triclinic	Triclinic	Triclinic	Triclinic	Triclinic	Triclinic	Monoclinic	Triclinic	Triclinic	Monoclinic	Triclinic	Monoclinic	Triclinic
Space Group	*P-1*	*P-1*	*P-1*	*P-1*	*P-1*	*P-1*	*P2_1_/a*	*P-1*	*P-1*	*P2_1_/n*	*P-1*	*P2_1_/c*	*P-1*
a (Å)	4.15	7.32	7.07	7.41	6.27	10.96	19.65	4.03	6.44	6.82	7.75	7.90	8.91
b (Å)	12.30	11.07	11.16	12.32	10.75	11.00	7.84	11.52	11.98	13.90	12.94	12.63	12.43
c (Å)	22.26	17.09	15.77	12.93	20.58	13.63	17.48	24.29	19.46	27.58	13.62	33.66	13.96
α (°)	98.4	95.9	75.3	96.5	103.0	87.9	90	86.6	76.0	90	91.4	90	99.8
β (°)	88.4	93.5	92.6	106.9	91.5	70.0	73.9	105.1	71.9	87.4	81.8	81.5	77.2
γ (°)	89.2	98.7	77.9	105.1	96.2	79.5	90	89.0	97.6	90	89.7	90	111.2
V (Å^3^)	1123	1359	1171	1082	1341	1519	2586	1085	1350	2613	1351	3322	1400.1
Z, Z’	2, 1	2, 1	2, 1	2, 1	2, 1	2, 1	4, 1	2, 1	2, 1	4, 1	2, 1	4, 1	2, 1
V/Z (Å^3^)	561	680	585	541	670	759	646	543	675	653	675	830	700
V/Z_calc_^a^ (Å^3^)	551	680	571	551	659	741	646	551	670	652	676	828	690
ρ, g cm^−3^	1.333	1.300	1.335	1.333	1.297	1.342	1.338	1.333	1.314	1.314	1.301	1.285	1.304
Gof	30.4	20.4	20.8	26.8	36.9	34.5	43.4	15.1	20.1	31.4	36.8	24.9	19.0
Onset T, °C	-	80.8	160.6 ^b^	-	130.5	113.6	173.8	-	130.4	147.7	162.6	129.1	-
To form	-	VI	VII	I	IXa, I	Xa	I	-	XIVab, I	I	I	NMP1	I
Dec. Onset T, °C	249.3	-	-	245.6	246.6	-	239.0	248.8	248.6	250.1	249.2	-	230.7 ^b^

^a^ Values calculated from the statistical analysis approach described in Hoffmann [15]; ^b^ complex event.

## Data Availability

The data presented in this study are available on request from the corresponding author. The data are not publicly available due to industrial protection issues.

## Sample Availability

Not available.
